# Identification, Serotyping and Antimicrobial Resistance in *Ornithobacterium rhinotracheale* Isolates from Turkeys in Poland Between 2016 and 2022

**DOI:** 10.3390/ani16020191

**Published:** 2026-01-08

**Authors:** Marek Blanda, Marcin Śmiałek, Joanna Kowalczyk, Olimpia Kursa

**Affiliations:** 1 Nativet Veterinary Clinic, ul. Piotrowskiego 10e, 10-692 Olsztyn, Poland; blanda6@interia.pl; 2 Department of Poultry Diseases, Faculty of Veterinary Medicine, University of Warmia and Mazury, ul. Oczapowskiego 13, 10-719 Olsztyn, Poland; joanna.kowalczyk@uwm.edu.pl; 3 SLW Biolab Veterinary Laboratory, ul. Grunwaldzka 62, 14-100 Ostróda, Poland; 4 Department of Bacteriology and Bacterial Animal Diseases, National Veterinary Research Institute, Al. Partyzantów 57, 24-100 Puławy, Poland; olimpia.kursa@piwet.pulawy.pl

**Keywords:** antibiotics, *Ornithobacterium rhinotracheale*, resistance, serotypes, turkeys

## Abstract

Respiratory diseases in poultry caused by *Ornithobacterium rhinotracheale* represent a significant challenge from both therapeutic and economic perspectives. Treatment of them, which uses antimicrobial agents, improperly contributes to the emergence and dissemination of multidrug-resistant strains within avian populations. The present study, based on *O. rhinotracheale* isolates (*n* = 733) obtained from the respiratory tract, joints, and organs of turkeys in Poland between 2016 and 2022, was conducted to assess the antimicrobial resistance of the strains and identify their serotypes. All *O. rhinotracheale* isolates were tested for antimicrobial susceptibility using the disk-diffusion method. The results of this study indicated that amoxicillin/clavulanic acid, doxycycline, florfenicol, and lincomycin/spectinomycin were the most effective agents against the isolates from turkeys. The findings showed that serotype I was the most prevalent among the tested turkey strains in Poland and was the serotype to which 29% of the *O. rhinotracheale* isolates affiliated.

## Simple Summary

The increasing number of multidrug-resistant microorganisms that spread within bird populations is a major problem in veterinary medicine. In our study, we assessed the prevalence and antibiotic resistance of *Ornithobacterium rhinotracheale* strains isolated from turkeys. The obtained results indicate relatively stable antimicrobial susceptibility profiles of isolates.

## 1. Introduction

In large-scale poultry production, bacterial infections continue to pose serious diagnostic and therapeutic challenges, while also causing substantial economic losses. One of the bacterial pathogens responsible for disease in birds is *Ornithobacterium rhinotracheale* (ORT), the etiological agent of ornithobacteriosis. This bacterium was first described in the 1990s and has since spread almost worldwide [1,2]. *Ornithobacterium rhinotracheale* occurs in farmed birds such as chickens, turkeys, ducks and geese, but has also been isolated from wild species including pigeons, gulls, falcons, and rooks [3,4,5]. Because of the pantropic nature of *O. rhinotracheale*, symptoms such as respiratory disease, arthritis, and tendonitis are observed during infection; systemically, decreased egg production and increased mortality are noted [6,7,8]. The severity and duration of the disease vary considerably and depend on factors such as the condition of the birds, coexisting infections, environmental influences, and hygiene standards [9].

To date, 18 serotypes (A–R) have been identified using serological methods among ORT strains. Serotype A is dominant among isolates obtained from chickens, whereas turkey isolates show greater diversity and most often belong to serotypes A, B, and E. However, it should be noted that there is a certain correlation between the geographical origin of isolates and their serotype. For example, serotype C was for a long time isolated only from birds in Africa and the USA [9,10,11]. It is now known that all serotypes are distributed worldwide, with the dominance of individual ones confirmed in specific geographic regions [9,10,11].

The treatment of bacterial infections caused by ORT is rather challenging because the susceptibility of individual isolates to antimicrobial agents varies. This variability is often associated, among other factors, with the geographic region from which a particular strain originates and with the local antibiotic selection pressure. Numerous studies have demonstrated the effectiveness of amoxicillin, ampicillin, erythromycin, neomycin, penicillin, spectinomycin, and tylosin against ORT [9]. Current therapeutic guidelines in Poland recommend amoxicillin with clavulanic acid and doxycycline as first-line chemotherapeutic agents for the treatment of infections caused by this bacterium, and list florfenicol, lincospectin, and sulfonamides combined with trimethoprim as second-line agents [12]. However, adherence to guidelines should be discretionary, because targeted antibiotic therapy based on susceptibility testing of specific isolates is possible, and is intended to significantly reduce the development of bacterial antibiotic resistance and thereby improve the effectiveness of treatment.

Expanding the information base underlying poultry treatment decisions is supportive of better treatment effectiveness. With this aim, the present studies were undertaken to determine the antibiotic susceptibility of and the prevalence of serotypes among *Ornithobacterium rhinotracheale* strains isolated from turkeys from farms located in Poland between 2016 and 2022.

## 2. Materials and Methods

### 2.1. Sample Collection and Bacterial Isolation

Samples for the detection of ORT were collected between 2016 and 2022 from poultry farms (*n* = 633) in six provinces of Poland (Table 1). The material consisted of tracheal swabs taken antemortem from turkeys showing respiratory symptoms such as sneezing, coughing, swelling of the infraorbital sinuses, and nasal discharge, as well as from birds that had died from disease or were euthanized on farms because their disease symptoms associated with ORT were advanced.

In the laboratory, samples were collected from dead birds which in life had exhibited respiratory signs or with evident joint lesions. Swabs were taken from the infraorbital sinuses and air sacs and the hock and hip joints, and tissue samples were collected aseptically from the trachea, lungs, brain and heart.

All samples were cultured on tryptic soy agar (TSA PS 22-500, Graso Biotech, Starogard Gdański, Poland) supplemented with sheep blood (SL0160-500, BioMaxima S.A., Lublin, Poland) and incubated under an atmosphere enriched with 5% CO_2_ at 37 ± 1 °C for 48 ± 3 h. Colonies suspected of being ORT were first identified manually (based on phenotypic traits) and subsequently analyzed using matrix-assisted laser desorption/ionization–time-of-flight mass spectrometry (MALDI-TOF MS; Biotyper, Bruker, Billerica, MA, USA). The obtained spectra were compared with reference spectra, which in most cases enabled unambiguous species identification.

### 2.2. Antibiotic Susceptibility Testing

All isolated and identified ORT strains were tested for antimicrobial susceptibility using the disk-diffusion method. The analyses were carried out on Mueller–Hinton agar (REF 116, Graso Biotech, Starogard Gdański, Poland) supplemented with sheep blood (SL0160-500, BioMaxima S.A., Lublin, Poland) using diagnostic disks supplied by Oxoid (Basingstoke, UK) and Liofilchem S.r.l. (Roseto degli Abruzzi, Italy). Sensitivity to 15 active substances was tested. The Oxoid antibiotics were amoxicillin (AML, 25 µg), amoxicillin with clavulanic acid (AMC, 30 µg), colistin (CL, 10 µg), doxycycline (DO, 30 µg), enrofloxacin (ENR, 5 µg), florfenicol (FFC, 30 µg), flumequine (FLM/UB, 30 µg), lincomycin/spectinomycin (LS, 109 µg), neomycin (N, 30 µg), oxytetracycline (OT, 30 µg), sulfamethoxazole/trimethoprim (SXT, 25 µg), and sulfonamides (S3, 300 µg; comprising sulfamerazine 82.83 µg, sulfathiazole 114.59 µg, and sulfadiazine 116.72 µg). The Liofilchem antibiotics were tylosin (TY, 30 µg), lincomycin (MY, 15 µg), and tiamulin (T, 30 µg).

A suspension of bacteria was prepared from a 24 h culture of ORT grown on TSA (TSA PS 22-500, Graso Biotech, Starogard Gdański, Poland) with sheep blood (SL0160-500, BioMaxima S.A., Lublin, Poland) in sterile deionized water to a density of 1 on the McFarland scale. The inoculum was spread over the agar surface, and antibiotic disks were placed on the medium. Plates were incubated for 16–18 h at 33–37 °C in an atmosphere enriched with 5% CO_2_.

After incubation, the diameters of the inhibition zones were measured in transmitted light using a caliper, and the results were entered into computer software that converted the values into antimicrobial susceptibility interpretations. The obtained results were classified as resistant, intermediate, or susceptible. Interpretation was performed according to the guidelines of the Clinical and Laboratory Standards Institute (CLSI) [13]. For quality control of the test, the *Streptococcus pneumoniae* ATCC 49619 reference strain was used.

### 2.3. Serotyping of ORT Strains

The serotyping of *Ornithobacterium rhinotracheale* strains (*n* = 411) was performed in an external laboratory. The laboratory of the Institute of Poultry Diseases in the Department of Veterinary Medicine at the Freie Universität Berlin undertook this using the agar gel precipitation test with reference antisera (A–L). Isolates that did not react with any of the available antisera were classified as non-typeable.

### 2.4. Statistical Analysis

To determine statistical differences between the numbers of ORT strains isolated from field samples that were resistant to particular antimicrobial agents, the *chi-square* (χ^2^) test was used. The analysis included all isolates obtained between 2016 and 2022 (the total number of analyzed strains was the sum from all seven years). Statistical analysis was performed using the Statistica 13.1 software package (StatSoft Polska, Kraków, Poland), and differences were considered statistically significant at *p* ≤ 0.05.

## 3. Results

A total of 773 strains were isolated and identified as ORT using MALDI-TOF MS. The majority of isolates (86%) originated from the trachea and infraorbital sinuses, while the remaining strains were obtained from the lungs and air sacs (9%), brain and heart (3%), and hock and hip joints (2%).

The antibiotic susceptibility of all tested strains is presented in Table 2. Analysis of the obtained data indicated that AMC (100% susceptible strains), LS (99%), FFC (98%), and DO (97%) demonstrated the highest efficacy against *O. rhinotracheale* isolates. Conversely, CL and N were ineffective against almost all strains (99%).

Table 3 presents the statistical significance of differences between the number of ORT strains resistant to the tested antibiotics. Statistically significant differences (*p* ≤ 0.05) were found in the number of strains when comparing their resistance to CL, ENR, FLM/UB, MY, S3, and T with all other antibiotics. No statistically significant differences were observed between the number of strains resistant to AML and the number resistant to OT (*p* = 0.1299), or between AMC and DO, FFC, or LS (*p* = 0.0582, *p* = 0.1018, and *p* = 0.0582, respectively). Similarly, no significant differences were noted between FLM/UB and CL or N (*p* = 0.1111 and *p* = 0.3134), between CL and N (*p* = 0.5633), or between FFC and LS (*p* = 0.7622).

**Figure 1:**
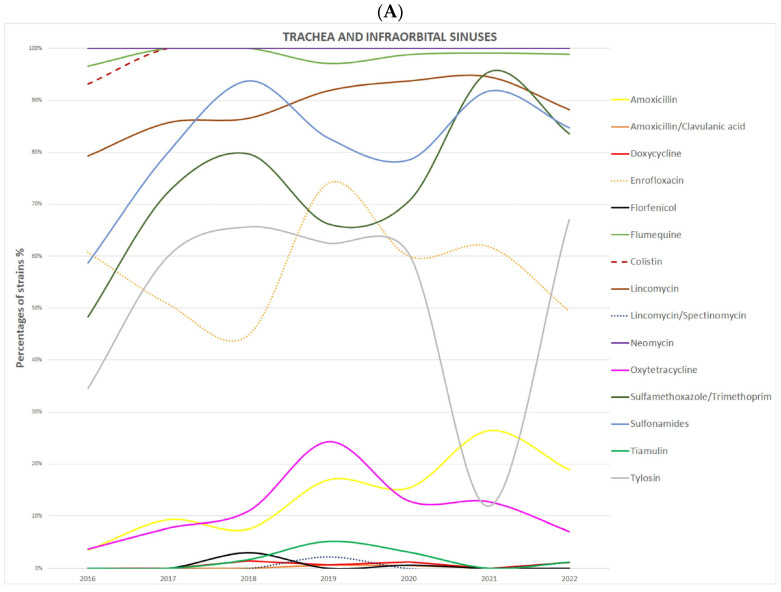

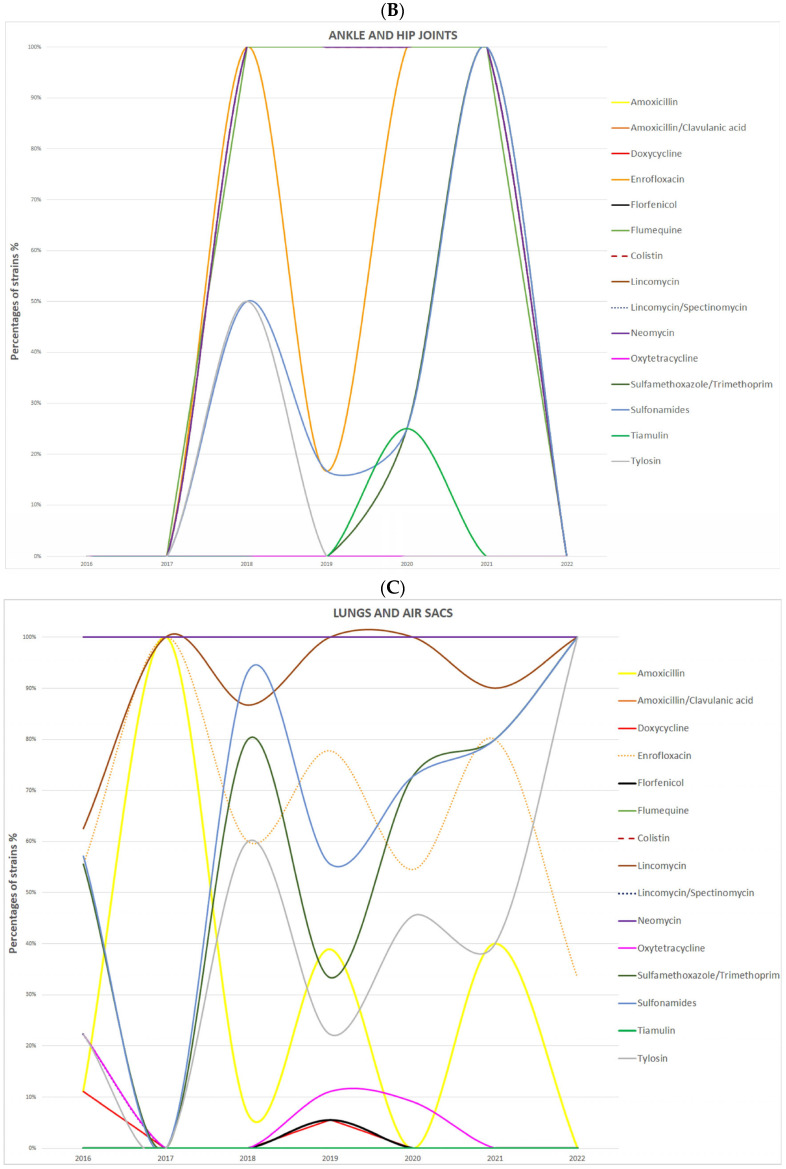
(**A**–**C**) Percentage of antibiotic-resistant *Ornithobacterium rhinotracheale* strains in the consecutive years between 2016 and 2022. (**A**)—Trachea and infraorbital sinuses; (**B**)—Ankle and hip joints; (**C**)—Lungs and air sacs.

To determine the trend of changes in ORT resistance to individual antibiotics over the years 2016–2022, the percentages year by year of resistant strains by anatomical site sampled for each active compound were plotted (Figure 1). The percentage of strains resistant to CL, N, and FLM/UB remained stable throughout the analyzed period across all sample types, including tracheal, infraorbital sinus, joint, and lung isolates. An increase in the percentage of multidrug-resistant isolates was observed in 2021 compared to 2019 and 2020. At the same time, a marked decrease in the proportion of strains resistant to TY was recorded in 2021 among isolates obtained from the trachea, infraorbital sinuses, lungs, and air sacs.

**Figure 2:**
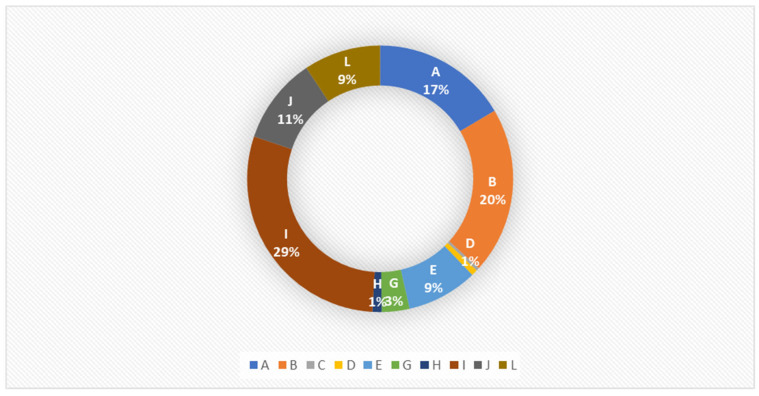
Percentage of serotypes of *Ornithobacterium rhinotracheale* isolated between 2016 and 2022.

Out of the 773 ORT strains isolated, 411 were subjected to serotyping. Of these, 267 samples were typable, while the remaining isolates were classified as non-typable because they did not react with diagnostic antisera. Figure 2 presents the percentage distribution of individual serotypes among all analyzed samples. Serotype I was the most frequently isolated during the study period, accounting for approximately 29% of all isolates. Serotypes A and B were also common, representing 17% and 20% of isolates, respectively.

Table 4 gives the percentage distribution of ORT serotypes in the examined samples according to the site of isolation (trachea and infraorbital sinus, hock and hip joints, and lungs and air sacs). Among isolates from the trachea and infraorbital sinuses, serotypes I (29%) and B (22%) predominated, whereas in isolates from the joints and lungs, serotypes A and I (30%) were the most common. Interestingly, in isolates from the hock and hip joints, serotypes B and L, present in samples from the respiratory tract, were not detected at all.

## 4. Discussion

Respiratory diseases in poultry caused by *Ornithobacterium rhinotracheale* represent a serious therapeutic as well as economic problem. Improper use of antibiotics, including metaphylactic, prophylactic, and empirical treatments administered without performing antimicrobial susceptibility testing of isolates beforehand, contributes to an increase in the number of multidrug-resistant microorganisms that subsequently spread within bird populations.

Our study, based on ORT strains isolated from turkeys between 2016 and 2022, indicates relatively stable antimicrobial susceptibility profiles over the years. However, an increase in multidrug-resistant isolates was observed in 2021, which is noteworthy considering the data published by Śmiałek et al. [14] showing a reduction in antibiotic use in turkey flocks during the same period. It is worth noting, however, that the cited studies [14] had limited territorial coverage and therefore may not fully reflect the real extent of antibiotic use in turkey populations. The increase in the number of multidrug-resistant ORT strains may result from the excessive use of antibiotics for treating bacterial infections other than ORT, exerting selective pressure on field strains. On the other hands, the Thirteenth ESVAC Report, in 2022, sales of antimicrobial veterinary medicinal products (VMPs) for food-producing animals in 31 European countries reached 4458.1 tonnes, including 838.3 tonnes in Poland alone [15]. Although a global decline in the sales of antimicrobial agents has been observed, this trend is less pronounced in Poland and is evident only in certain drug groups.

According to the literature, the mechanisms of resistance acquisition in *O. rhinotracheale* are not yet fully understood, but they are presumed to involve plasmid- and gene-mediated mutations [16,17]. Considering the above, our observations may also result from the spread of resistance genes within and between bacterial species [18], although this hypothesis needs further study.

Chin and Droual [19] and Hafez [20] reported almost 100% susceptibility of isolates to AML, chloramphenicol, and tetracycline, and resistance to ENR, N, and SXT. These findings are partly consistent with our results, in which 99% of isolates were resistant to N, 71% to SXT, and 60% to ENR. On the other hand, only 69% of Polish *O. rhinotracheale* isolates were susceptible to AML. In the Netherlands, the USA, and Hungary, resistance to ampicillin, ceftiofur, tetracycline, and ENR has been reported, while in Iranian isolates, susceptibility to ENR and tetracycline was observed [6,21,22,23,24]. These data partially correspond with our findings, showing that Polish ORT isolates exhibited the highest susceptibility to AMC, LS, FFC, and DO. In contrast, Hassan et al. [25] reported susceptibility of isolates to DO and N, to which the majority of Polish isolates were resistant. Analysis of global data on antimicrobial susceptibility clearly shows a high degree of variability, which can be explained by geographical region, genetic diversity of isolates, and differences in therapeutic strategies used in various areas.

Serotyping of ORT allows for classification of bacterial isolates into serotypes A–R. According to available data, the dominant serotype in chickens worldwide is A, whereas in turkeys, greater diversity is observed, with serotypes A, B, and E alternating by region as the most common [9,10,11,20]. Our results indicate that serotype I predominates in the turkey population in Poland, accounting for nearly 30% of all tested isolates and surpassing serotypes A and B. Another indication of serotype diversity in turkeys is that isolates from hock and hip joint swabs were identified as serotype C, which for a long time had been reported only in birds from Africa and the USA [9,10,11]. The isolation of serotype C in Poland is, among other factors, a result of the national epizootic situation regarding avian influenza, which necessitated the import of birds from other markets, including Canada. This import also occurs independently of Poland’s official status with regard to notifiable diseases. These findings confirm a changing trend in the distribution of individual *O. rhinotracheale* serotypes depending on geographic region and time, reflecting the ongoing spread and evolution of the bacterium.

Additionally, in rapid agglutination tests, cross-reactions were observed between serotypes B and A and between serotypes I and L. Other studies have also demonstrated cross-reactions between serotypes A, E, and I, but not between any other and serotype C, which indicates certain limitations of this diagnostic method [7,9,10].

Currently, immunoprophylaxis is considered the most effective strategy for controlling ORT infections in poultry flocks. Various vaccines have been developed and evaluated, including live, inactivated, and recombinant preparations, differing in bacterial inactivation methods and adjuvants. The best results have been obtained with oil-adjuvanted inactivated vaccines, which induced the strongest immune response and significantly reduced disease lesions in birds [26,27].

Commercial vaccines available globally contain serotypes A, B, and C; however, their applicability and efficacy on poultry farms are often limited because vaccine components and the serotypes currently dominant in specific regions are ever less homologous. Furthermore, in some countries, including Poland, no commercial vaccines against *O. rhinotracheale* are currently registered. Therefore, autogenous vaccines, whose antigenic composition is tailored to the current epizootic situation of a specific flock, farm, or region, play an important role in ORT prevention. In the approach using these vaccines, each farm is treated individually, taking into account regional circumstances, environmental pressure, and infection history.

Autogenous vaccines are widely used in turkey flocks, and a two-dose vaccination regimen effectively protects birds against the adverse effects of ORT infection. Moreover, previous studies have shown that the use of autogenous vaccines can substantially reduce antibiotic use in poultry flocks—on average by approximately 19% over three years [14].

## 5. Conclusions

In summary, strategies for preventing infectious diseases in poultry, including ornithobacteriosis, should be based on animal welfare, effective biosecurity, and immunoprophylaxis [28]. Although our study suggests that the susceptibility of the analyzed ORT isolates, collected in Poland between 2016 and 2022, to most commonly used antimicrobials remains stable, it should be noted that it may vary depending on the geographic region from which a particular strain originates and the local antibiotic selection pressure. Proper flock management and an optimal vaccination program tailored to the specific farm can significantly reduce bacterial infections such as *Ornithobacterium rhinotracheale* and, consequently, the need for antibiotic therapy. At the same time, reducing antibiotic use in poultry production may substantially contribute to slowing or even preventing the development of bacterial resistance to antimicrobial agents.

## Figures and Tables

**Table 1:** Regional distributions of isolates.

	Isolates n (%)							
Provinces	2016	2017	2018	2019	2020	2021	2022	n
**Dolnośląskie**			2 (50)		2 (50)			4
**Lubelskie**					1 (50)	1 (50)		2
**Mazowieckie**	20 (10)	16 (8)	20 (10)	41 (21)	50 (25)	26 (13)	26 (13)	199
**Podlaskie**		1 (12.5)		1 (12.5)		6 (75)		8
**Pomorskie**	2 (11)	5 (28)	2 (11)	6 (33)	1 (6)	2 (11)		18
**Warmińsko-mazurskie**	15 (4)	36 (9)	37 (9)	76 (19)	113 (28)	75 (19)	49 (12)	402

**Table 2:** Antimicrobial susceptibility of *Ornithobacterium rhinotracheale* strains (*n* = 773) isolated from turkeys between 2016 and 2022.

Antimicrobial	n	R	R%	I	I%	S	S%
**Amoxicillin**	773	123	16	115	15	535	69
**Amoxicillin/Clavulanic acid**	773	1	0	1	0	771	100
**Doxycycline**	773	6	1	19	2	748	97
**Enrofloxacin**	773	463	60	276	36	34	4
**Florfenicol**	773	5	1	7	1	761	98
**Flumequine**	773	758	98	10	1	6	1
**Colistin**	773	769	99	0	0	4	1
**Lincomycin**	773	691	89	4	1	78	10
**Lincomycin/Spectinomycin**	773	6	1	1	0	766	99
**Neomycin**	773	763	99	0	0	10	1
**Oxytetracycline**	773	102	13	67	9	604	78
**Sulfamethoxazole/Trimethoprim**	773	551	71	96	12	126	16
**Sulfonamides**	773	613	79	91	12	69	9
**Tiamulin**	773	40	5	68	9	665	86
**Tylosin**	773	455	59	81	10	237	31

n—number of samples; R—resistant strains; R%—percentage of resistant strains; I—intermediate strains; I%—percentage of intermediate strains; S—susceptible strains; S%—percentage of susceptible strains.

**Table 3:** Evaluation of statistical significance between the numbers of *Ornithobacterium rhinotracheale* isolates resistant to the tested antibiotics between 2016 and 2022. *p* < 0.05 indicates pairs of antibiotics that show statistically significant difference in the resistance rate.

	Amoxicillin n = 123	Amoxicillin/ Clavulanic Acid n = 1	Doxycycline n = 6	Enrofloxacin n = 463	Florfenicol n = 5	Flumequine n = 758	Colistin n = 769	Lincomycin n = 691	Lincomycin/ Spectinomycin n = 6	Neomycin n = 763	Oxytetracycline n = 102	Sulfamethoxazole/ Trimethoprim n = 551	Sulfonamides n = 613	Tiamulin n = 40	Tylosin n = 455
**Amoxicillin n = 123**		*p* < 0.001	*p* < 0.001	*p* < 0.001	*p* < 0.001	*p* < 0.001	*p* < 0.001	*p* < 0.001	*p* < 0.001	*p* < 0.001	*p* = 0.1299	*p* < 0.001	*p* < 0.001	*p* < 0.001	*p* < 0.001
**Amoxicillin/** **Clavulanic acid n = 1**			*p* = 0.0582	*p* < 0.001	*p* = 0.1018	*p* < 0.001	*p* < 0.001	*p* < 0.001	*p* = 0.0582	*p* < 0.001	*p* < 0.001	*p* < 0.001	*p* < 0.001	*p* < 0.001	*p* < 0.001
**Doxycycline** **n = 6**				*p* < 0.001	*p* = 0.7622	*p* < 0.001	*p* < 0.001	*p* < 0.001	*p* = 1.000	*p* < 0.001	*p* < 0.001	*p* < 0.001	*p* < 0.001	*p* < 0.001	*p* < 0.001
**Enrofloxacin n = 463**					*p* < 0.001	*p* < 0.001	*p* < 0.001	*p* < 0.001	*p* < 0.001	*p* < 0.001	*p* < 0.001	*p* < 0.001	*p* < 0.001	*p* < 0.001	*p* = 0.6787
**Florfenicol** **n = 5**						*p* < 0.001	*p* < 0.001	*p* < 0.001	*p* = 0.7622	*p* < 0.001	*p* < 0.001	*p* < 0.001	*p* < 0.001	*p* < 0.001	*p* < 0.001
**Flumequine n = 758**							*p* = 0.111	*p* < 0.001	*p* < 0.001	*p* = 0.3134	*p* < 0.001	*p* < 0.001	*p* < 0.001	*p* < 0.001	*p* < 0.001
**Colistin** **n = 769**								*p* < 0.001	*p* < 0.001	*p* = 0.5633	*p* < 0.001	*p* < 0.001	*p* < 0.001	*p* < 0.001	*p* < 0.001
**Lincomycin n = 691**									*p* < 0.001	*p* < 0.001	*p* < 0.001	*p* < 0.001	*p* < 0.001	*p* < 0.001	*p* < 0.001
**Lincomycin/** **Spectinomycin** **n = 6**										*p* < 0.001	*p* < 0.001	*p* < 0.001	*p* < 0.001	*p* < 0.001	*p* < 0.001
**Neomycin** **n = 763**											*p* < 0.001	*p* < 0.001	*p* < 0.001	*p* < 0.001	*p* < 0.001
**Oxytetracycline** **n = 102**												*p* < 0.001	*p* < 0.001	*p* < 0.001	*p* < 0.001
**Sulfamethoxazole/** **Trimethoprim n = 551**													*p* < 0.001	*p* < 0.001	*p* < 0.001
**Sulfonamides** **n = 613**														*p* < 0.001	*p* < 0.001
**Tiamulin** **n = 40**															*p* < 0.001
**Tylosin** **n = 455**															

**Table 4:** The percentage of serotypes *Ornithobacterium rhinotracheale* isolated from the trachea and infraorbital sinuses, joints, lung and air sacs between 2016 and 2022.

	SEROTYPE (%)									
	A	B	C	D	E	G	H	I	J	L
**TRACHEA AND SUPRAORBITAL SINUSES**	15	22	0	1	9	4	1	29	10	10
**ANKLE AND HIP JOINTS**	29	0	14	0	14	0	0	29	14	0
**LUNGS AND AIR SACS**	30	13	0	0	4	0	0	30	17	4

## Data Availability

Data are contained within the article. The datasets used and/or analyzed during the current study are available from the corresponding author on reasonable request.

## Abbreviations

The following abbreviations are used in this manuscript:

ORT	*Ornithobacterium rhinotracheale*
AML	Amoxicillin
AMC	Amoxicillin/Clavulanic acid
CL	Colistin
DO	Doxycycline
ENR	Enrofloxacin
FFC	Florfenicol
FLM/UB	Flumequine
LS	Lincomycin/Spectinomycin
MY	Lincomycin
N	Neomycin
OT	Oxytetracycline
SXT	Sulfamethoxazole/Trimethoprim
S3	Sulfonamides
T	Tiamulin
TY	Tylosin
